# Endostatin inhibits VEGF-A induced osteoclastic bone resorption *in vitro*

**DOI:** 10.1186/1471-2474-7-56

**Published:** 2006-07-13

**Authors:** Annina Sipola, Katri Nelo, Timo Hautala, Joanna Ilvesaro, Juha Tuukkanen

**Affiliations:** 1 Dept. of Anatomy and Cell Biology, PO Box 5000, FIN-90014 University of Oulu, Finland; 2 Department of Internal Medicine, PO Box 5000, FIN-90014 University of Oulu, Finland

## Abstract

**Background:**

Endostatin is a C-terminal fragment of collagen XVIII which is a component of basement membranes with the structural properties of both collagens and proteoglycans. Endostatin has a major role in angiogenesis which is intimately associated with bone development and remodeling. Signaling between the endothelial cells and the bone cells, for example, may have a role in recruitment of osteoclastic precursor cells. Our study aims at exploring a possibility that endostatin, either as a part of basement membrane or as a soluble molecule, may control osteoclastogenesis and osteoclastic bone resorption *in vitro*.

**Methods:**

Rat pit formation assay was employed in order to examine the effect of endostatin alone or in combination with vascular endothelial growth factor-A (VEGF-A) on bone resorption *in vitro*. Effect of these agents on osteoclast differentiation *in vitro *was also tested. Osteoclastogenesis and the number of osteoclasts were followed by tartrate resistant acid phosphatase (TRACP) staining and resorption was evaluated by measuring the area of excavated pits.

**Results:**

Endostatin inhibited the VEGF-A stimulated osteoclastic bone resorption, whereas endostatin alone had no effect on the basal resorption level in the absence of VEGF-A. In addition, endostatin could inhibit osteoclast differentiation *in vitro *independent of VEGF-A.

**Conclusion:**

Our *in vitro *data indicate that collagen XVIII/endostatin can suppress VEGF-A induced osteoclastic bone resorption to the basal level. Osteoclastogenesis is also inhibited by endostatin. The regulatory effect of endostatin, however, is not critical since endostatin alone does not modify the basal bone resorption.

## Background

The development and continuous remodeling of the skeleton demand a tightly regulated balance between the bone-forming and bone-resorbing processes. One of the key components of bone remodeling is constant development of vasculature. Vasculature is required for transport of nutrients and precursor cells, such as precursors of chondroclasts and osteoclasts, to the renewing bone tissue. Angiogenesis has also been shown to be crucial for the replacement of cartilage by bone during skeletal growth and regeneration. Vascular endothelial growth factor-A (VEGF-A), produced by hypertrophic chondrocytes during endochondral bone formation, stimulates controlled invasion of chondroclasts into the cartilage [1,2]. VEGF-A is also a chemoattractant for endothelial cells and regulates the growth plate vascularisation of metaphyseal bone.

Invasion of osteoclasts into hypertrophic cartilage requires presence of VEGF-A [3] which binds with high affinity to two tyrosine kinase receptors, Flt-1 and Flk-1 [4]. VEGF-A may attract osteoclast precursor cells that are recruited from hematopoietic tissue. It has been reported that VEGF-A is potentially a monocyte chemoattractant [5]. Monocytes express Flt-1, but not Flk-1 [6]. In response to VEGF-A, macrophages derived from Flt-1 mutant mice indicated deranged chemotaxis [7] demonstrating that Flt-1 may mediate the migration of monocyte/macrophage lineages. Mature osteoclasts have been shown to express Flt-1 and Flk-1 on their cells surface [1,8-12] and both of these receptors may mediate the VEGF-A effect on bone formation and bone resorption [8]. Recently published data clearly indicates that VEGF-A is important both in endochondral and intramembranous ossification [13,14]. Precise mechanisms of the recruitment of osteoclast precursors into the site of bone resorption, however, remain unclear.

Endostatin, a known antagonist for VEGF-A, is 20 kDa C-terminal fragment of collagen XVIII found in basal membranes. Endostatin, that binds to αv- and α5-integrins [15], inhibits endothelial cell proliferation and it may inhibit angiogenesis and tumor growth [16]. Resorbing osteoclasts have been shown to express at least αv-integrins [17-19], creating a potential for endostatin to control osteoclast function. Role of endostatin on bone resorption or osteoclast differentiation, however, is not established.

Aim of our study was to further define possible role of endostatin and VEGF-A on osteoclasts *in vitro*. In order to investigate the direct effects of these angiogenic and antiangiogenic substances on osteoclast mediated bone resorption, we used classical resorption pit assay and osteoclast differentiation assay of bone marrow hematopoietic stem cells. We were, for the first time, able to demonstrate that endostatin has a significant regulatory role on both osteoclastic bone resorption and osteoclastogenesis *in vitro*.

## Methods

### Osteoclast isolation and culture

The procedure for the isolation and culture of osteoclasts described earlier by Boyde *et al*. and by Chambers *et al*. [20,21] was modified slightly and has been described in detail previously [22]. Briefly, mechanically harvested osteoclasts from the long bones of 1- or 2-day-old Sprague-Dawley rat pups were allowed to attach to ultrasonicated bovine cortical bone slices (0.125 cm^2 ^or 0.5 cm^2^). After 30 minutes, the nonattached cells were rinsed away, and the remaining cells on the bone slices were cultured in Dulbecco's modified Eagle's medium (α-MEM) buffered with 20 mM HEPES and containing 0.84 g of sodium bicarbonate/liter, 2 mM L-glutamine, 100 IU of penicillin/ml, 100 μg of streptomycin/ml and 7–10% heat-inactivated fetal calf serum (FCS), at +37°C (5% CO_2 _and 95% air). The cells were divided into ten groups: the control group had α-MEM-FCS as their culture medium with no added substances (later referred as control), the VEGF-A-treated cells had 100 ng/ml, 50 ng/ml or 10 ng/ml VEGF-A in α-MEM-FCS (later referred as VEGF). The endostatin groups had 0.02, 0.2 or 2 μg/ml endostatin in α-MEM-FCS (later referred as ENDO) and the last groups had both VEGF-A and endostatin added into the complete culture media (later referred as VEGF+ENDO). The last groups had 100 ng/ml VEGF-A and 2 μg/ml endostatin, 50 ng/ml VEGF-A and 0,2 μg/ml endostatin or 10 ng/ml VEGF-A and 0,02 μg/ml endostatin. The cells were allowed to grow for 48 h, after which the bone slices were fixed with freshly made refrigerated 3% paraformaldehyde (PFA) and 2% sucrose in phosphate-buffered saline (PBS) for 10 minutes at room temperature. The data shown in this manuscript is gathered from three independent experiments, each of which gave identical results.

### Osteoclast differentiation assay

The procedure for osteoclast differentiation described earlier by Takahashi et al. [23] was slightly modified. Mouse (C57BL/6, 8–12 weeks) bone marrow cells were isolated from mouse long bones by using syringe. After incubation at +37°C for 2 hours non-attached cells were collected from petri dish. Mouse bone marrow cells were seeded in 24-well plates containing sonicated cortical bovine bone slices (0.125–0.5 cm^2^) at a concentration of 1 × 10^6 ^cells/well. Cells on the bone slices were cultured in four groups: control, VEGF (100 ng/ml) and ENDO (2 μg/ml) and VEGF + ENDO respectively. The control group was cultured in α-MEM medium (Sigma, UK) with 10% heat-inactivated fetal calf serum (FCS), 2 mM L-glutamin, 100 IU/ml penicillin, 10 μg/ml streptomycin and 20 mM Hepes which medium will be later referred to as α-MEM-FCS. Cells were cultured in 500 μl α-MEM containing 30 ng/ml RANKL (Peprotech Ec., UK) and 10 ng/ml M-CSF (R&D Systems). Half of the medium was replaced every 3rd day. Cells were cultured at +37°C (5% CO2, 95% air) for 7 days, after which the cultures were stopped by fixing the cells with 3% paraformaldehyde (PFA)/2% sucrose in PBS.

### TRACP staining

Bone cells were cultured for 48 hours in resorption pit assay and 7 days in the differentiation assay. After the culture period, bone slices were fixed as described above. The cells were stained for tartrate-resistant acid phosphatase (TRACP), a commonly accepted marker of osteoclasts [24] using a Sigma TRACP kit (no. 386-A, Sigma Chemicals, St. Louis, MO) according to the manufacturer's instructions. To visualize the nuclei, the cells were incubated with the DNA-binding fluorochrome Hoechst 33258 (1 mg/ml stock diluted 1:800 in PBS, Sigma Chemical Co, St. Louis, MO) for 10 minutes at room temperature. The numbers of multinucleated TRACP-positive cells on each bone slice were counted, using a Nikon Eclipse 600 microscope and Nikon Plan Fluor 20×/0.50 objective with an appropriate filterset.

### Area measurement of excavated pits

Prior to the staining of the pits, total detachment of the cells from the bone slices was ensured by wiping the cellular surface of the slices with a soft brush. The pits were stained with peroxidase-conjugated wheat germ agglutinin-lectin (WGA; 20 μg/ml, Sigma Chemical Co., St. Louis, MO) for 20 minutes, washed with PBS, and incubated for 5 minutes in diaminobenzidine (0,5 mg/ml) +0,03% H_2_O_2_. Morphometric analysis of the resorption pits was performed with an MCID image analyzer, utilizing M_2 _software (Imaging Research Inc., Brock University, Ontario, Canada).

### Statistical analysis

Data presented are the results of at least two independent experiments. Values are the mean ± SEM. Significance was calculated using one-way ANOVA followed by Student's t-test with statistical package Origin 6.0. The p-values less than 0.05 were considered as significant.

## Results

### Bone resorption assay

**Figure 1:**
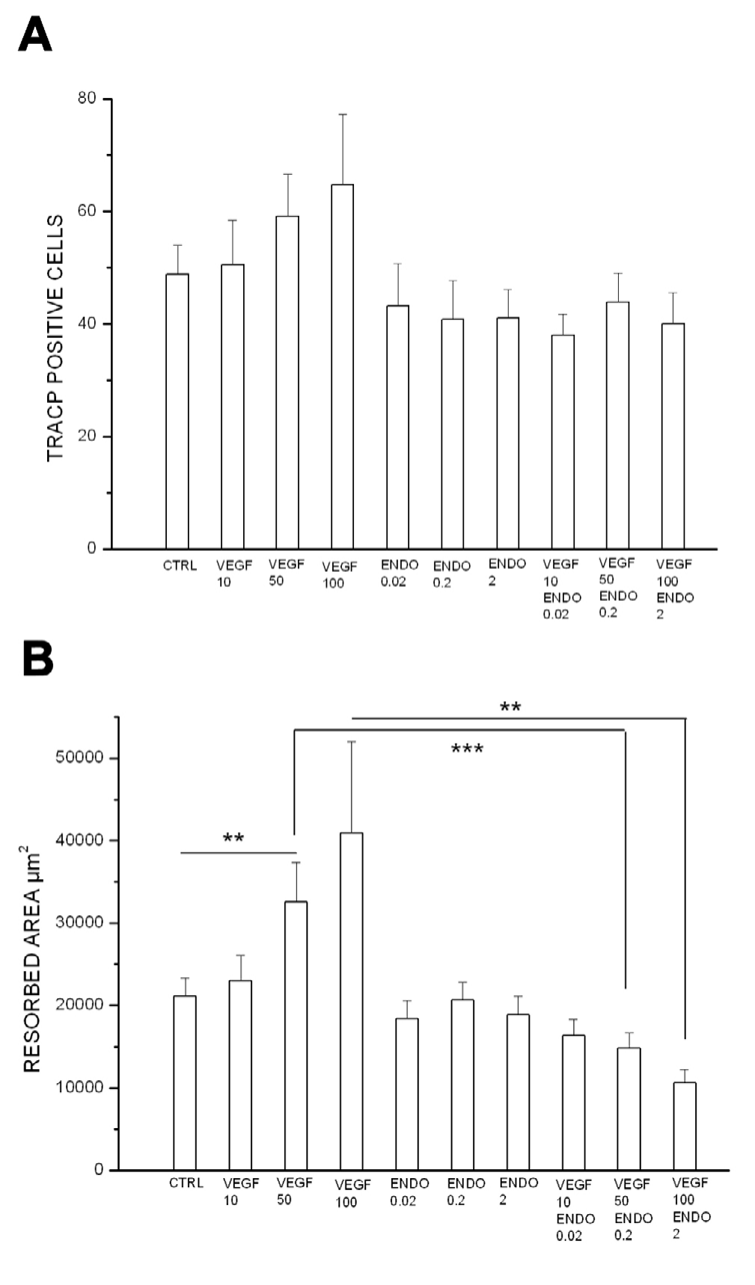
**Number of TRACP+ multinuclear cells and the activity of bone resorption in pit assay**. Rat bone cells were cultured for 48 hours with no added substances (**CONTROL**), vascular endothelial growth factor A (**VEGF **ng/ml), endostatin (**ENDO **μg/ml) or both VEGF-A and endostatin (**VEGF **ng/ml +**ENDO **μg/ml). (**A**) Survival rate of osteoclasts after different treatments was measured by counting the numbers of TRACP-positive cells with two or more nuclei indicating osteoclasts. No significant differences in the number of osteoclasts could be seen. (**B**) Osteoclastic bone resorption could be visualized with WGA-lectin stain and total resorbed areas from the different cultures were measured. VEGF-A treatment induced a clear stimulus, whereas endostatin caused no effect on the resorbed area. Addition of both VEGF-A and endostatin caused a statistically significant decrease in the total resorbed area when compared to the VEGF-A-induced resorption. (**VEGF **ng/ml, **ENDO **μg/ml)

The number of osteoclasts after 48 hours in each sample group was counted by analyzing multinuclear TRACP-positive cells in the resorption assay. No significant differences in the number osteoclasts were found between the different groups (Fig 1A) indicating that none of the treatments was toxic to these cells.

**Figure 2:**
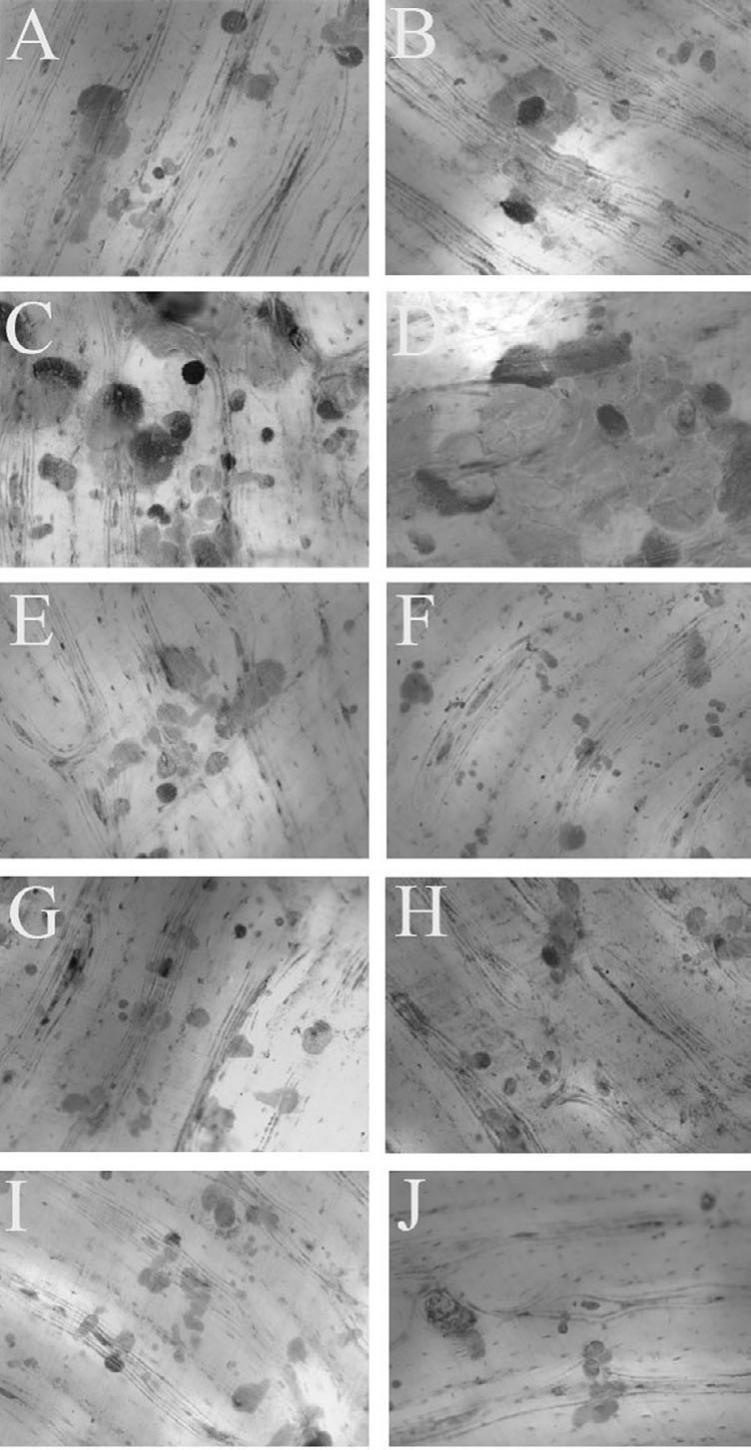
**Typical appearance of the bone slices in the pit assay**. Rat bone cells were cultured on bovine bone slices with different treatments. After 48 hours, cells were wiped off and resorbed bone area was visualized with image analyzer system. Bone slices after **(A) **Control **(B) **VEGF-A 10 ng/ml **(C) **VEGF-A 50 ng/ml **(D) **VEGF-A 100 ng/ml **(E) **Endostatin 0.02 μg/ml **(F) **Endostatin 0.2 μg/ml **(G) **Endostatin 2 μg/ml **(H) **VEGF-A 10 ng/ml and Endostatin 0.02 μg/ml **(I) **VEGF-A 50 ng/ml and Endostatin 0.2 μg/ml **(J) **VEGF-A 100 ng/ml and Endostatin 2 μg/ml treatments. Scale bar 100 μm.

The total resorbed area was significantly increased in response to VEGF-A treatment and the effect was dose dependent (Fig. 1B). Endostatin alone had no inhibitory effect on the resorbed area when compared to the basal level of osteoclastic bone resorption. When both endostatin and VEGF-A were supplemented, the stimulatory effect by VEGF-A on bone resorption was halted to the basal level. The resorption activity was even below basal level with the highest concentrations (p < 0.05 in ctrl vs. VEGF100 and endostatin). Thus, endostatin could block the VEGF-A-induced stimulatory effect on osteoclastic bone resorption. These results are also demonstrated in figure 2 in which representative bone slices are shown.

### Osteoclast differentiation

**Figure 3:**
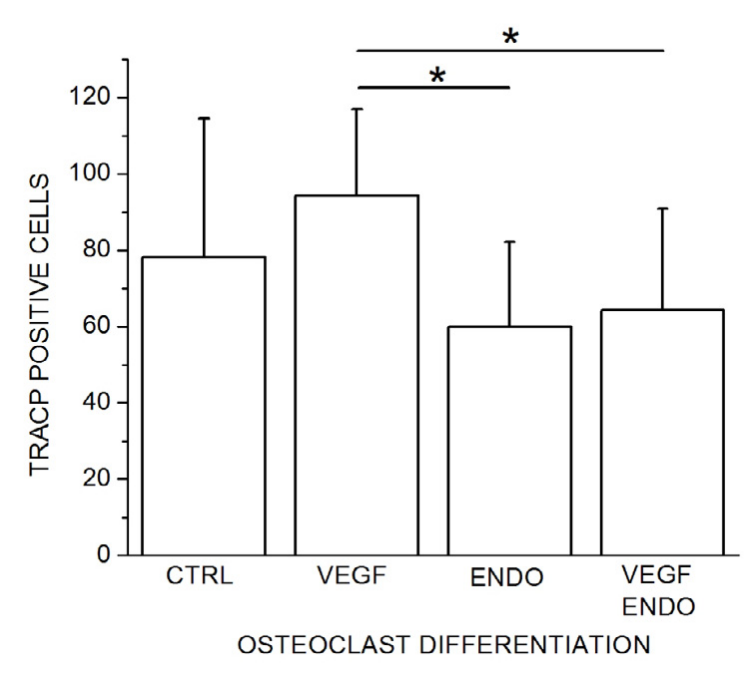
**The number of TRACP+ multinuclear cells**. Mouse (C57BL/6, 8–12 weeks) bone marrow cells were isolated from mouse long bones and seeded in 24-well plates on bovine bone slices. Cells on the bone slices were cultured in four groups: control, VEGF (100 ng/ml) and ENDO (2 μg/ml) and VEGF + ENDO respectively. The control group was cultured in α-MEM with 10% FCS containing 30 ng/ml RANKL and 10 ng/ml M-CSF for 7 days, after which the cultures were stopped by fixing the cells with 3% paraformaldehyde (PFA)/2% sucrose in PBS.

In the presence of RANKL and M-CSF the bone marrow hematopoietic cells differentiate into osteoclasts which can be recognized by positive TRACP-stain and multinuclear appearance. Since the basal resorption was not affected by endostatin in the pit assay, we conducted an assay to investigate if endostatin could inhibit the differentiation of osteoclasts. We added VEGF-A to the medium containing M-CSF and RANKL in order to achieve maximal differentiation stimulus (Fig. 3). Endostatin was able to abolish the maximal osteoclastogenesis and to reduce the number of TRACP+ cells in the presence of VEGF, RANKL and M-CSF or RANKL and M-CSF only.

## Discussion

Bone remodeling is initiated by recruitment of osteoclast precursor cells from bone marrow or circulating monocytes. Homing of these precursors through the vascular wall is required for successful bone turnover. Intimate contact of the bone remodeling site to adjacent vascular sinusoids indicates interplay between the vascular endothelium and the bone cells. Co-culture of osteoblasts and endothelial cells stimulate the osteoblasts to secrete VEGF-A and to express RANKL, which is crucial for osteoclastogenesis [25]. VEGF-A is a chemoattractant for osteoclasts and lack of VEGF-A halts invasion of osteoclasts into hypertrophic cartilage through an MMP-9 dependent mechanism [3]. The similar kinetics and signaling pathways of RANKL and VEGF-A as chemoattractants differ from those of M-CSF [26]. The significance of VEGF-A in osteoclastogenesis is clearly demonstrated in osteopetrotic mice with a mutation in the M-CSF gene [27-29]. The mice have severe deficiency of osteoclasts, monocytes, and peritoneal macrophages. Injections of purified recombinant human M-CSF (rhM-CSF) compensate for these deficiencies in the mutant mice [30-33]. Interestingly, a single injection of rhVEGF-A can substitute M-CSF and cure osteopetrosis [8].

Significance of endostatin in bone has been demonstrated elegantly in the *ex vivo *metatarsal model in which fusion and migration of osteoclast precursors is required for the osteoclastic bone resorption [26]. Osteoclastic recruitment from periosteum can be inhibited by endostatin, leading to blocked dissolution of the calcified matrix in the developing marrow cavity. Endostatin completely inhibits VEGF-A and placental growth factor-2 mediated osteoclast invasion but has no effect on RANKL or M-CSF induced migration. The recruitment and invasion of osteoclast precursors can be disturbed by endostatin in the metatarsal model. Correspondingly, soluble Flt-1 blocks invasion of osteoclasts into the marrow cavity of developing long bones *in vivo *[8]. In our pit formation assay *in vitro*, mature osteoclasts already exist. Still, we see a clear reduction of VEGF-A-stimulated resorption in the endostatin treated pit assays. Thus, it is possible that endostatin may not only limit the migration of osteoclast precursors, but it may also inhibit the VEGF-A-induced osteoclast activation. Pufe et al. have detected similar influence of endostatin on hyaline articular cartilage. Endostatin alone had no effect on basal levels on the phosphorylation of ERK1/2, whereas co-incubation with endostatin blocked VEGF-induced ERK1/2 phosphorylation in immortalized human chondrocytes (C28/I2) [34,35].

Osteoclast precursor differentiation into mature osteoclasts is disturbed in the absence of VEGF-A [5]. VEGF-A also aids in the bone-resorbing function of osteoclasts [8,11]. Nakagawa and co-workers [11] have shown that the presence of VEGF-A enhances survival of pure rabbit osteoclasts and increases the resorbed bone area. In our resorption pit assay containing osteoblasts and osteoclasts, however, the number of TRACP-positive cells remained unchanged (Fig. 1A). The data show that the increased total resorbed area in our model (Fig. 1B) is not due to enhanced survival rate. The observed effect is more likely a result of a direct VEGF-A stimulation on osteoclastic bone resorption.

Combination of RANKL, M-CSF, and VEGF-A activated intense differentiation of osteoclasts in our study *in vitro*. RANKL and VEGF-A stimulate the osteoclast recruitment through the same signaling pathway [26] and these pathways were most likely fully stimulated in our cell cultures. Endostatin, however, inhibited osteoclastogenesis when compared to this maximal stimulus (achieved with the combination of M-CSF, RANKL, and VEGF-A). Interestingly, the inhibitory effect was also seen in the absence of VEGF-A. This result indicates that inhibition by endostatin cannot be solely explained as an antagonism of VEGF-A.

Endostatin did not inhibit basal bone resorption in our *in vitro *experiments suggesting that regulatory role of endostatin on osteoclasts may not be critical. In pathological conditions, however, endostatin may have a significant position. Endostatin is normally found in serum [36,37] and a possible therapeutic role for endostatin has been studied in cancer with promising results [38-40]. Endostatin could also be considered as a physiological inhibitor of inflammation induced neovascularization in arthritis patients. VEGF-A levels are elevated in arthritis and thus osteoclastic bone resorption is enhanced. Recently a clinical trial on patients with rheumatoid arthritis showed increased serum endostatin levels after a single injection of a TNF-α antibody. In addition, endostatin overexpression inhibited development of arthritis in the joints of TNF-transgenic mice [41]. Our *in vitro *data support the possibility that endostatin could be used as a physiological agent to inhibit the osteoclast activity in inflammatory arthritis. Further *in vivo *studies in animal models may be considered to explore that possibility.

## Conclusion

Our *in vitro *data indicate that collagen XVIII/endostatin can suppress VEGF-A induced osteoclastic bone resorption to the basal level. Osteoclastogenesis is also inhibited by endostatin. The regulatory effect of endostatin, however, is not critical since endostatin alone does not modify the basal bone resorption.

## Competing interests

The author(s) declare that they have no competing interests.

## Authors' contributions

AS, KN and JI have performed the cell cultures and their examination including the statistical analysis and together with TH and JT participated in development of the experiments. All authors have read and approved the final manuscript.

## Pre-publication history

The pre-publication history for this paper can be accessed here:
